# COVID-19 mass vaccination – an illustration of the impact of syringe choice on the effectiveness of mass vaccination campaigns

**DOI:** 10.1080/22221751.2022.2048968

**Published:** 2022-03-12

**Authors:** Roseline Mazet, Luc Choisnard, Julien Leenhardt, Olivier Epaulard, Etienne Brudieu, Pierrick Bedouch

**Affiliations:** a Pharmacy Unit, Grenoble University Hospital, Grenoble, France; b University Grenoble Alpes DPM, Saint Martin d'Hères, France; c Infectious Diseases Department, Grenoble University Hospital, Grenoble, France; d Groupe de recherche en infectiologie clinique, University Grenoble Alpes, Grenoble, France; e University Grenoble Alpes TIMC, Grenoble, France

**Keywords:** Mass vaccination, COVID-19, medical device, public health, vaccination capacity

## Abstract

In order to optimise the operational implementation of mass vaccination policies, it is critical to consider not only the supply of vaccines as well as each element of the vaccination process. This study, which was conducted in a vaccination center clearly shows how the choice of a syringe reference used during the COVID-19 vaccination campaign influences the number of vaccine doses available. The results appear to be closely related to the type of vaccine used (COMIRNATY® and SPIKEVAX®). In this context, the choice of the right reference of syringe has major economic and organisational consequences on a global scale.

How critical is the drug, the choice of medical device, operator and their combination in massive public health action? Our work illustrates by the management of the COVID-19 vaccination problem in France.

The coronavirus disease 2019 (COVID-19) pandemic has dramatically affected the lives of billions of people with considerable health, societal and economic consequences worldwide. The spread of the severe acute respiratory syndrome coronavirus 2 (SARS-CoV-2) has persisted despite the implementation of effective, restrictive public health measures [1]. The deployment of COVID-19 vaccines offers a hope for the alleviation of the immense health, societal and economic tolls. Real-life data confirmed that licensed COVID-19 vaccines are safe and highly effective in providing protection against symptomatic and severe COVID-19, and also against asymptomatic SARS-CoV-2 infection and transmission [2]. In this context, some countries around the world have implemented “vaccine passports” to provide a strong incentive for vaccination, thereby promoting individual and herd immunity [3–5]. In order to immunize as many people as possible in the shortest possible time, mass vaccination centres were created worldwide. In our area, the mass vaccination centre run by Grenoble University Hospital (CHUGA) has the capacity to vaccinate 5,000 people a day. On 31 August 2021, 1,354,882 doses had been administered and 185,559 vials had been dispensed in this vaccination centre so far. At this scale, the control of each step of the vaccination process is paramount. Each factor can have a major economic and organizational impact, which can be considerable on a national and global scale.

According to their summary of product characteristics, the COMIRNATY® (BioNTech/Pfizer; Bnt162b2) and the SPIKEVAX® (Moderna) vaccines are packaged to obtain respectively 6 and 10 doses per vial, respectively [6, 7]. However, due to overfilling by manufacturers, the number of doses that can actually be extracted from the vials is in practice higher: 7 doses with the COMIRNATY® (BioNTech/Pfizer) and 12 doses with the SPIKEVAX® (Moderna) vaccines. Nevertheless, feedback from vaccination centres reported an irregular number of doses extracted from vaccines vials after a change of syringe reference: 6 or 7 doses with the COMIRNATY® (BioNTech/Pfizer) and 11 or 12 doses with the SPIKEVAX® (Moderna) vaccines. Meanwhile, the non-implementation of the 7th dose with COMIRNATY® (BioNTech/Pfizer) vaccines and of the 12th dose with SPIKEVAX® (Moderna) vaccines could have a major impact on vaccination capacity. In coordination with *Santé Publique France*, a government agency “responsible” in charge of, among others, all supply related to COVID-19 vaccination, we investigated this syringe issue in light of the number of extractable doses, to understand its origin, and to resolve it.

In order to get as close as possible to real operational conditions, the impact of the syringe model used on the number of extractable doses from COMIRNATY® and SPIKEVAX® vaccine vials was studied *in situ* in CHUGA mass vaccination centre. This study focused on feedback provided by vaccination centres incriminating the syringes used and it was not intended to compare differences between vaccination centres or to assess the individual practices of vaccination operators. The two syringes tested (HPMT and ZIMD v1) are routinely used in the vaccination centre by two experienced vaccination operators (Table S1). Results are reported in Figure 1.

**Figure 1.**
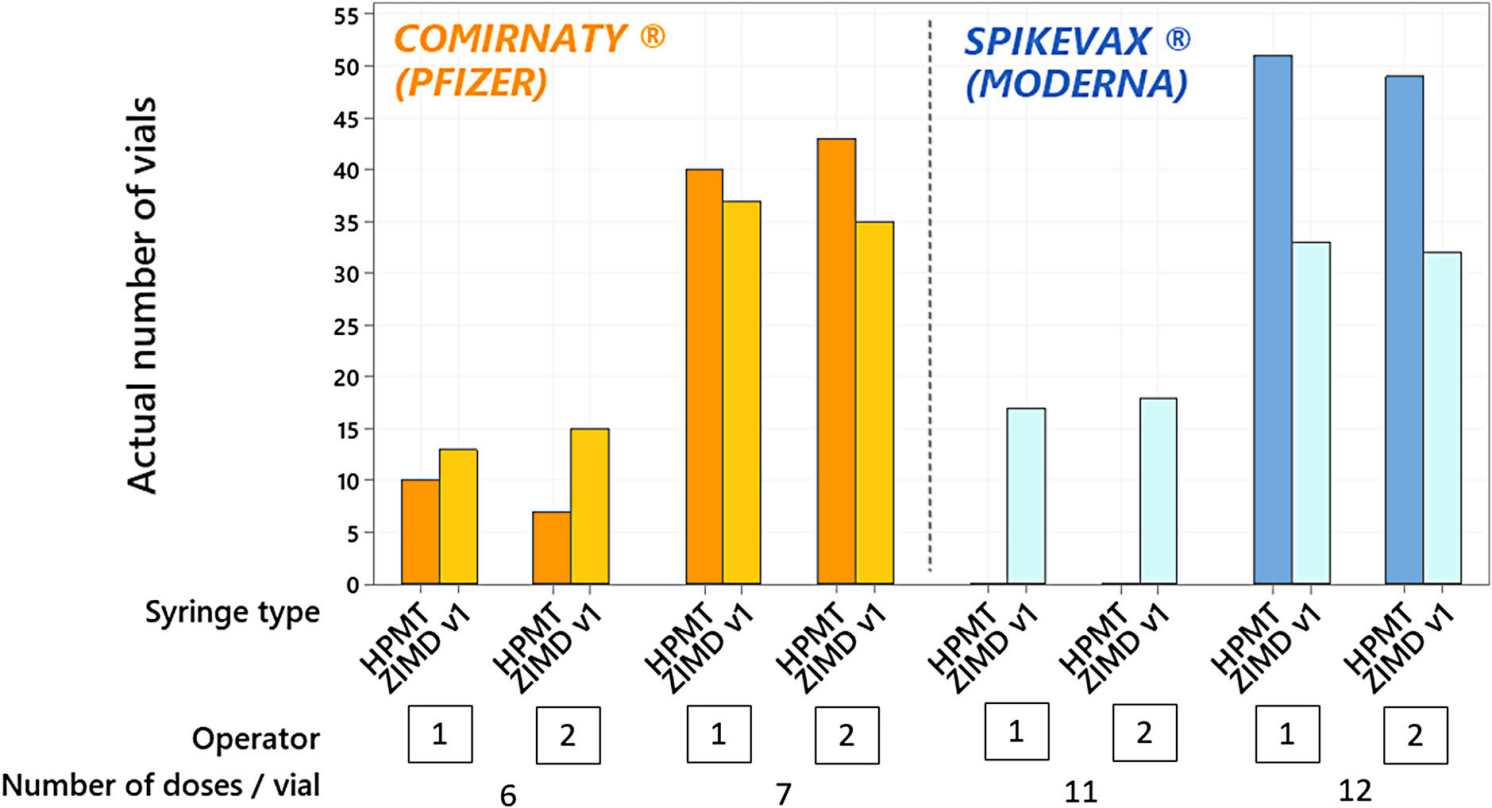
Number of vials capable of extracting 6 or 7 doses with the COMIRNATY® and 11 or 12 doses with the SPIKEVAX® vaccines, considering the type of syringe used (ZIMDv1and HPMT) and the operators (*n* = 2). *N* = 100 vials / vaccine supplier / type of syringe.

As illustrated in the figure, the number of extractable doses per vaccine’s vial, regardless of the vaccine (COMINATY® or SPIKEVAX®), is independent of the vaccination operator.

As far as COMIRNATY® vial is concerned, the proportions of vials from which the operator succeeded in extracting 7 doses were 0.72 for ZIMD v1 and 0.83 for HPMT, this difference is not significant. Conversely, for the SPIKEVAX® vial, the proportions of vials from which the operator succeeded in extracting 12 doses per vial were 0.65 for ZIMD v1 and 1.00 for HPMT (*p*-value < 0.001). Based on the results, the syringe model is shown to impact only the number of doses extracted from SPIKEVAX® vials. An average of 12 doses per vial is extracted with HPMT syringes; whereas an average of 11.65 doses per vial are extracted with ZIMD v1 syringes.

In order to understand the observed difference, we evaluated the actual volume extracted with the two types of syringes to produce a 0.5 mL of SPIKEVAX® vaccine dose (Table S2). The average volumes actually withdrawn from the two types of syringes to produce a vaccinal dose are respectively 0.495mL and 0.501 mL for HPMT and ZIMD v1 (*n* = 80 per syringe). This difference is statistically significant (*P*-value < 0.001). Considering the filling volume of the SPIKEVAX® vial, this difference, even if small, could partly explain why it is not possible to obtain 12 doses with the ZIMD v1 syringes. Note that both HPMT and ZIMD v1 are CE certified according to NF EN ISO 7886–1 and therefore cannot be considered as defective [8].

Several factors could further account for the difference in the number of doses extracted from SPIKEVAX® vial, such as: the skills of the vaccination operators, the filling volumes of the vaccination vials and /or the design of the vial and the septum.

However, in the present, the change of type of syringe used seems to be particularly critical. Considering that the purchase of this device is often entails a national choice, this issue of unfulfilled doses due to a change of syringe reference can have a major impact in terms of health, ethics and economy both on a national and international level. The non-implementation of this 12th dose of the SPIKEVAX® (Moderna) vaccine could have a major impact on vaccination capacity. In view of the importance of the number of vaccinations carried out by the CHUGA, the loss is estimated at approximately 2,700 unfulfilled doses, or 225 vials of SPIKEVAX® (Moderna), i.e. a value of €50,000 ($60,000).

The supplier of ZIMD v1 was informed and was quick to modify the syringe. The first tests performed with ZIMD v2 (same reference) show an average volume of 0.491 ml (Table S2). This volume may tend to solve the observed problem (Figure S1). The effective improvement induced by the change of medical device on vaccination capacity must obviously be confirmed in situ in the vaccination centres.

As we have observed in vaccination centres, the number of doses of vaccine actually delivered does not depend exclusively on the volume of vaccine vials filled. This work highlights the relevance of the choice of each medical device used in mass public health action, especially syringes, so as to optimize the performance of vaccination centres in the current health context. Naturally, many other factors may influence the number of doses extractable per vial of vaccine in addition to this rapid operational gain related to a change of syringe type. In the longer term, a study including several vaccination centres and a larger panel of operators with different levels of expertise could be very informative.

## Supplementary Material

Supplemental Material
