# Supplementary material for: COVID-19 mass vaccination – an illustration of the impact of syringe choice on the effectiveness of mass vaccination campaigns
Source: Emerg Microbes Infect. 2022 Mar 12;11(1):804–6. doi: 10.1080/22221751.2022.2048968 (PMC8920360; doi:10.1080/22221751.2022.2048968)
Supplement: Supplemental Material [file TEMI_A_2048968_SM3756.docx]

**Supplementary Materials for**

**COVID-19 mass vaccination - an illustration of the impact of medical devices on the efficiency of mass vaccination campaigns**

**Materials And Methods**

## Observational study of vaccine number of doses extracted per vaccine vial

The evaluation was performed *in situ* at the mass vaccination center managed by the Grenoble Alpes University Hospital, France. The following considerations were taken into account in the study design:

- In the context of the pandemic, each dose intentionally not extracted from a vaccine vial corresponds to one unvaccinated patient. For obvious ethical reasons, we therefore limited the number of vaccine vials involved in the study to the strict minimum necessary to achieve the study objectives.

- The study focused mainly on the impact analysis of the choice of syringe used, as this was the factor most frequently mentioned by professionals in the field. This factor could also be a quick win.

- The number of operators was reduced for organizational reasons in the vaccination centres and to ensure sufficient statistical power for the study of the type of syringe.

Finally, the effective number of extractable doses per vaccine’s vial was studied for 2 syringe references: ZIMD v1 (1 mL ref.: 215000 - Zhejiang INI Medical Devices Co, Ltd, China) and HPMT (1 mL ref. 210000 - Hunan Pingan Medical Technology Co, Ltd, China). This work was conducted by 2 professional operators. Operator 1 is a 40-year-old nurse (level of study: bachelor +3) with more than 15 years of experience in her activity. Operator 2 is a 42-year-old nurse manager (bachelor + 5) with more than 15 years of experience in her activity. Before starting the study, both operators were trained on arrival at the vaccination centre in the same way as all other staff involved in the vaccination process. This training consisted of:

- A documentary debriefing on the methods of reconstitution and use of COMIRNATY - Pfizer / BioNTech and MODERNA.

- A short period of mentoring by experienced colleagues (3 half days).

Study was carried out on 100 vials of COMIRNATY® (BioNTech/Pfizer) and SPIKEVAX® (Moderna) vaccines per syringe type and per operator (table S1). Figure 1 and results were analyzed by Khi-2 procedure using Minitab v19 software.

***Accuracy study of the extracted volume from syringes to produce a 0.5 mL dose of SPIKEVAX® vaccine (Moderna)***

In this study, the use of real SPIKEVAX® vaccine solution was excluded for obvious ethical reasons. Required vaccine dose corresponds to a volume of 0.5mL. We sought to know the actual volume extracted by the two syringe references (HPMT and ZIMD v1). This volume was evaluated by gravimetric measurement on 0.9% sodium chloride injectable solution (KABIPAC®, BBraun, France). The actual average sampling volume was determined on 80 tests per syringe references (table S2). Figure S1 and results were analyzed by Mann-Whitney procedure using Minitab.

**Supplementary figure**


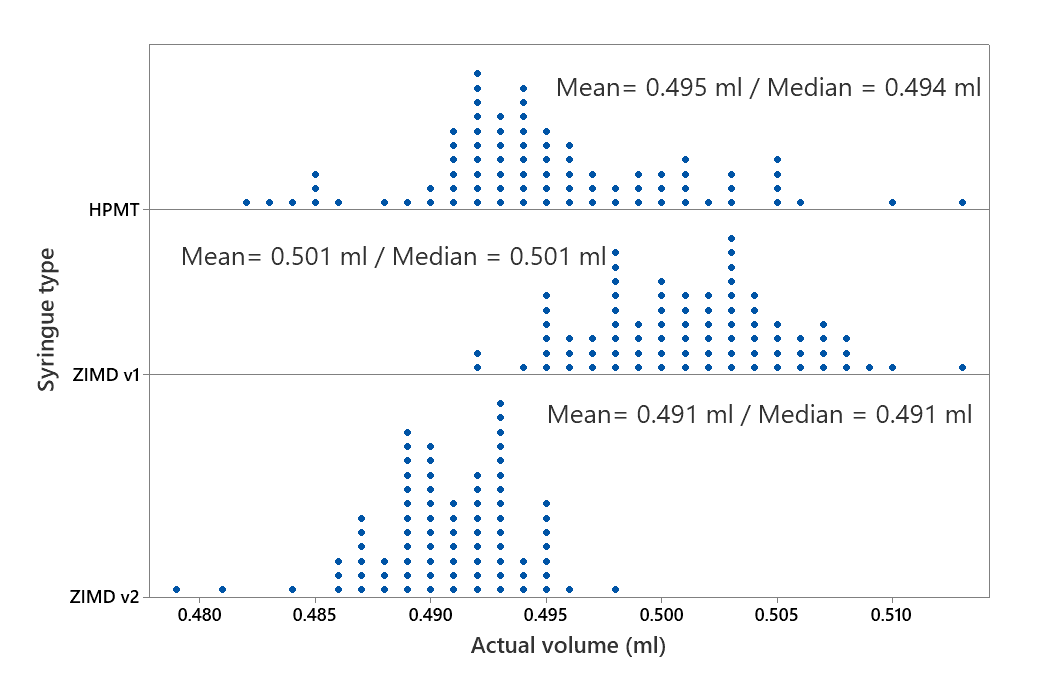


**Figure S1**: Dotplot of actual value of volumes drawn per syringe to deliver a dose of 0.5 mL (NaCl 0.9%) as a function of the type of syringe (HPMT, ZIMED v1 or ZIMED v2). N=80 per syringe type.

**Supplemental tables**

**Table S1**: Number of vials capable of extracting 6 or 7 doses with the COMIRNATY® and 11 or 12 doses with the SPIKEVAX® vaccines, considering the type of syringe used (ZIMDv1and HPMT) and the operators (n=2). N=100 vials / vaccine supplier / type of syringe.

| Syringe type | Vial number | Vaccine | Operator | Actual number of doses / vial |
| --- | --- | --- | --- | --- |
| ZIMD v1 | Vial 1 | Pfizer | 1 | 7 |
| ZIMD v1 | Vial 2 | Pfizer | 2 | 6 |
| ZIMD v1 | Vial 3 | Pfizer | 1 | 6 |
| ZIMD v1 | Vial 4 | Pfizer | 2 | 7 |
| ZIMD v1 | Vial 5 | Pfizer | 1 | 7 |
| ZIMD v1 | Vial 6 | Pfizer | 2 | 6 |
| ZIMD v1 | Vial 7 | Pfizer | 2 | 6 |
| ZIMD v1 | Vial 8 | Pfizer | 1 | 7 |
| ZIMD v1 | Vial 9 | Pfizer | 1 | 7 |
| ZIMD v1 | Vial 10 | Pfizer | 2 | 7 |
| ZIMD v1 | Vial 11 | Pfizer | 1 | 7 |
| ZIMD v1 | Vial 12 | Pfizer | 1 | 7 |
| ZIMD v1 | Vial 13 | Pfizer | 2 | 7 |
| ZIMD v1 | Vial 14 | Pfizer | 1 | 7 |
| ZIMD v1 | Vial 15 | Pfizer | 2 | 7 |
| ZIMD v1 | Vial 16 | Pfizer | 2 | 7 |
| ZIMD v1 | Vial 17 | Pfizer | 1 | 7 |
| ZIMD v1 | Vial 18 | Pfizer | 1 | 6 |
| ZIMD v1 | Vial 19 | Pfizer | 2 | 7 |
| ZIMD v1 | Vial 20 | Pfizer | 1 | 7 |
| ZIMD v1 | Vial 21 | Pfizer | 2 | 6 |
| ZIMD v1 | Vial 22 | Pfizer | 1 | 7 |
| ZIMD v1 | Vial 23 | Pfizer | 2 | 7 |
| ZIMD v1 | Vial 24 | Pfizer | 1 | 6 |
| ZIMD v1 | Vial 25 | Pfizer | 1 | 7 |
| ZIMD v1 | Vial 26 | Pfizer | 2 | 6 |
| ZIMD v1 | Vial 27 | Pfizer | 2 | 6 |
| ZIMD v1 | Vial 28 | Pfizer | 2 | 6 |
| ZIMD v1 | Vial 29 | Pfizer | 1 | 6 |
| ZIMD v1 | Vial 30 | Pfizer | 2 | 7 |
| ZIMD v1 | Vial 31 | Pfizer | 1 | 7 |
| ZIMD v1 | Vial 32 | Pfizer | 2 | 7 |
| ZIMD v1 | Vial 33 | Pfizer | 1 | 7 |
| ZIMD v1 | Vial 34 | Pfizer | 2 | 7 |
| ZIMD v1 | Vial 35 | Pfizer | 1 | 7 |
| ZIMD v1 | Vial 36 | Pfizer | 2 | 7 |
| ZIMD v1 | Vial 37 | Pfizer | 1 | 7 |
| ZIMD v1 | Vial 38 | Pfizer | 2 | 7 |
| ZIMD v1 | Vial 39 | Pfizer | 1 | 7 |
| ZIMD v1 | Vial 40 | Pfizer | 2 | 7 |
| ZIMD v1 | Vial 41 | Pfizer | 1 | 7 |
| ZIMD v1 | Vial 42 | Pfizer | 1 | 7 |
| ZIMD v1 | Vial 43 | Pfizer | 2 | 7 |
| ZIMD v1 | Vial 44 | Pfizer | 1 | 7 |
| ZIMD v1 | Vial 45 | Pfizer | 2 | 7 |
| ZIMD v1 | Vial 46 | Pfizer | 1 | 7 |
| ZIMD v1 | Vial 47 | Pfizer | 2 | 7 |
| ZIMD v1 | Vial 48 | Pfizer | 2 | 6 |
| ZIMD v1 | Vial 49 | Pfizer | 1 | 7 |
| ZIMD v1 | Vial 50 | Pfizer | 2 | 7 |
| ZIMD v1 | Vial 51 | Pfizer | 1 | 7 |
| ZIMD v1 | Vial 52 | Pfizer | 2 | 7 |
| ZIMD v1 | Vial 53 | Pfizer | 1 | 7 |
| ZIMD v1 | Vial 54 | Pfizer | 2 | 6 |
| ZIMD v1 | Vial 55 | Pfizer | 1 | 7 |
| ZIMD v1 | Vial 56 | Pfizer | 2 | 7 |
| ZIMD v1 | Vial 57 | Pfizer | 1 | 7 |
| ZIMD v1 | Vial 58 | Pfizer | 2 | 7 |
| ZIMD v1 | Vial 59 | Pfizer | 1 | 6 |
| ZIMD v1 | Vial 60 | Pfizer | 2 | 7 |
| ZIMD v1 | Vial 61 | Pfizer | 1 | 7 |
| ZIMD v1 | Vial 62 | Pfizer | 2 | 7 |
| ZIMD v1 | Vial 63 | Pfizer | 1 | 7 |
| ZIMD v1 | Vial 64 | Pfizer | 2 | 7 |
| ZIMD v1 | Vial 65 | Pfizer | 1 | 7 |
| ZIMD v1 | Vial 66 | Pfizer | 2 | 7 |
| ZIMD v1 | Vial 67 | Pfizer | 1 | 7 |
| ZIMD v1 | Vial 68 | Pfizer | 2 | 6 |
| ZIMD v1 | Vial 69 | Pfizer | 1 | 7 |
| ZIMD v1 | Vial 70 | Pfizer | 2 | 7 |
| ZIMD v1 | Vial 71 | Pfizer | 1 | 7 |
| ZIMD v1 | Vial 72 | Pfizer | 1 | 6 |
| ZIMD v1 | Vial 73 | Pfizer | 2 | 7 |
| ZIMD v1 | Vial 74 | Pfizer | 1 | 7 |
| ZIMD v1 | Vial 75 | Pfizer | 2 | 7 |
| ZIMD v1 | Vial 76 | Pfizer | 1 | 7 |
| ZIMD v1 | Vial 77 | Pfizer | 2 | 6 |
| ZIMD v1 | Vial 78 | Pfizer | 1 | 6 |
| ZIMD v1 | Vial 79 | Pfizer | 2 | 7 |
| ZIMD v1 | Vial 80 | Pfizer | 1 | 7 |
| ZIMD v1 | Vial 81 | Pfizer | 2 | 7 |
| ZIMD v1 | Vial 82 | Pfizer | 1 | 6 |
| ZIMD v1 | Vial 83 | Pfizer | 2 | 7 |
| ZIMD v1 | Vial 84 | Pfizer | 1 | 6 |
| ZIMD v1 | Vial 85 | Pfizer | 2 | 7 |
| ZIMD v1 | Vial 86 | Pfizer | 1 | 6 |
| ZIMD v1 | Vial 87 | Pfizer | 2 | 6 |
| ZIMD v1 | Vial 88 | Pfizer | 1 | 7 |
| ZIMD v1 | Vial 89 | Pfizer | 2 | 6 |
| ZIMD v1 | Vial 90 | Pfizer | 1 | 7 |
| ZIMD v1 | Vial 91 | Pfizer | 2 | 7 |
| ZIMD v1 | Vial 92 | Pfizer | 1 | 7 |
| ZIMD v1 | Vial 93 | Pfizer | 2 | 7 |
| ZIMD v1 | Vial 94 | Pfizer | 2 | 6 |
| ZIMD v1 | Vial 95 | Pfizer | 1 | 6 |
| ZIMD v1 | Vial 96 | Pfizer | 2 | 6 |
| ZIMD v1 | Vial 97 | Pfizer | 1 | 6 |
| ZIMD v1 | Vial 98 | Pfizer | 2 | 7 |
| ZIMD v1 | Vial 99 | Pfizer | 1 | 6 |
| ZIMD v1 | Vial 100 | Pfizer | 2 | 7 |
| HPMT | Vial 101 | Pfizer | 1 | 7 |
| HPMT | Vial 102 | Pfizer | 2 | 6 |
| HPMT | Vial 103 | Pfizer | 1 | 7 |
| HPMT | Vial 104 | Pfizer | 1 | 7 |
| HPMT | Vial 105 | Pfizer | 2 | 6 |
| HPMT | Vial 106 | Pfizer | 1 | 7 |
| HPMT | Vial 107 | Pfizer | 2 | 7 |
| HPMT | Vial 108 | Pfizer | 1 | 7 |
| HPMT | Vial 109 | Pfizer | 2 | 7 |
| HPMT | Vial 110 | Pfizer | 1 | 7 |
| HPMT | Vial 111 | Pfizer | 2 | 7 |
| HPMT | Vial 112 | Pfizer | 1 | 7 |
| HPMT | Vial 113 | Pfizer | 2 | 7 |
| HPMT | Vial 114 | Pfizer | 1 | 7 |
| HPMT | Vial 115 | Pfizer | 2 | 6 |
| HPMT | Vial 116 | Pfizer | 2 | 7 |
| HPMT | Vial 117 | Pfizer | 1 | 6 |
| HPMT | Vial 118 | Pfizer | 1 | 7 |
| HPMT | Vial 119 | Pfizer | 2 | 7 |
| HPMT | Vial 120 | Pfizer | 1 | 6 |
| HPMT | Vial 121 | Pfizer | 2 | 7 |
| HPMT | Vial 122 | Pfizer | 2 | 7 |
| HPMT | Vial 123 | Pfizer | 1 | 7 |
| HPMT | Vial 124 | Pfizer | 2 | 7 |
| HPMT | Vial 125 | Pfizer | 1 | 7 |
| HPMT | Vial 126 | Pfizer | 2 | 7 |
| HPMT | Vial 127 | Pfizer | 1 | 7 |
| HPMT | Vial 128 | Pfizer | 2 | 6 |
| HPMT | Vial 129 | Pfizer | 1 | 7 |
| HPMT | Vial 130 | Pfizer | 2 | 7 |
| HPMT | Vial 131 | Pfizer | 1 | 7 |
| HPMT | Vial 132 | Pfizer | 2 | 7 |
| HPMT | Vial 133 | Pfizer | 1 | 7 |
| HPMT | Vial 134 | Pfizer | 1 | 6 |
| HPMT | Vial 135 | Pfizer | 2 | 7 |
| HPMT | Vial 136 | Pfizer | 1 | 7 |
| HPMT | Vial 137 | Pfizer | 2 | 7 |
| HPMT | Vial 138 | Pfizer | 1 | 7 |
| HPMT | Vial 139 | Pfizer | 2 | 7 |
| HPMT | Vial 140 | Pfizer | 2 | 7 |
| HPMT | Vial 141 | Pfizer | 1 | 7 |
| HPMT | Vial 142 | Pfizer | 2 | 7 |
| HPMT | Vial 143 | Pfizer | 2 | 7 |
| HPMT | Vial 144 | Pfizer | 1 | 7 |
| HPMT | Vial 145 | Pfizer | 2 | 7 |
| HPMT | Vial 146 | Pfizer | 1 | 7 |
| HPMT | Vial 147 | Pfizer | 2 | 7 |
| HPMT | Vial 148 | Pfizer | 2 | 7 |
| HPMT | Vial 149 | Pfizer | 1 | 7 |
| HPMT | Vial 150 | Pfizer | 2 | 7 |
| HPMT | Vial 151 | Pfizer | 1 | 7 |
| HPMT | Vial 152 | Pfizer | 2 | 7 |
| HPMT | Vial 153 | Pfizer | 1 | 6 |
| HPMT | Vial 154 | Pfizer | 1 | 7 |
| HPMT | Vial 155 | Pfizer | 2 | 6 |
| HPMT | Vial 156 | Pfizer | 1 | 7 |
| HPMT | Vial 157 | Pfizer | 2 | 7 |
| HPMT | Vial 158 | Pfizer | 1 | 7 |
| HPMT | Vial 159 | Pfizer | 2 | 7 |
| HPMT | Vial 160 | Pfizer | 1 | 7 |
| HPMT | Vial 161 | Pfizer | 2 | 7 |
| HPMT | Vial 162 | Pfizer | 1 | 7 |
| HPMT | Vial 163 | Pfizer | 2 | 7 |
| HPMT | Vial 164 | Pfizer | 1 | 7 |
| HPMT | Vial 165 | Pfizer | 2 | 7 |
| HPMT | Vial 166 | Pfizer | 1 | 7 |
| HPMT | Vial 167 | Pfizer | 2 | 7 |
| HPMT | Vial 168 | Pfizer | 1 | 6 |
| HPMT | Vial 169 | Pfizer | 2 | 7 |
| HPMT | Vial 170 | Pfizer | 1 | 6 |
| HPMT | Vial 171 | Pfizer | 2 | 7 |
| HPMT | Vial 172 | Pfizer | 1 | 7 |
| HPMT | Vial 173 | Pfizer | 2 | 7 |
| HPMT | Vial 174 | Pfizer | 1 | 6 |
| HPMT | Vial 175 | Pfizer | 1 | 7 |
| HPMT | Vial 176 | Pfizer | 2 | 7 |
| HPMT | Vial 177 | Pfizer | 2 | 7 |
| HPMT | Vial 178 | Pfizer | 2 | 7 |
| HPMT | Vial 179 | Pfizer | 1 | 7 |
| HPMT | Vial 180 | Pfizer | 2 | 7 |
| HPMT | Vial 181 | Pfizer | 1 | 6 |
| HPMT | Vial 182 | Pfizer | 1 | 7 |
| HPMT | Vial 183 | Pfizer | 2 | 7 |
| HPMT | Vial 184 | Pfizer | 1 | 7 |
| HPMT | Vial 185 | Pfizer | 2 | 7 |
| HPMT | Vial 186 | Pfizer | 1 | 7 |
| HPMT | Vial 187 | Pfizer | 2 | 6 |
| HPMT | Vial 188 | Pfizer | 1 | 7 |
| HPMT | Vial 189 | Pfizer | 2 | 7 |
| HPMT | Vial 190 | Pfizer | 2 | 7 |
| HPMT | Vial 191 | Pfizer | 1 | 7 |
| HPMT | Vial 192 | Pfizer | 2 | 6 |
| HPMT | Vial 193 | Pfizer | 1 | 7 |
| HPMT | Vial 194 | Pfizer | 2 | 7 |
| HPMT | Vial 195 | Pfizer | 1 | 6 |
| HPMT | Vial 196 | Pfizer | 2 | 7 |
| HPMT | Vial 197 | Pfizer | 1 | 6 |
| HPMT | Vial 198 | Pfizer | 2 | 7 |
| HPMT | Vial 199 | Pfizer | 1 | 7 |
| HPMT | Vial 200 | Pfizer | 1 | 7 |
| ZIMD v1 | Vial 1 | Moderna | 1 | 11 |
| ZIMD v1 | Vial 2 | Moderna | 2 | 12 |
| ZIMD v1 | Vial 3 | Moderna | 1 | 12 |
| ZIMD v1 | Vial 4 | Moderna | 2 | 12 |
| ZIMD v1 | Vial 5 | Moderna | 2 | 11 |
| ZIMD v1 | Vial 6 | Moderna | 1 | 12 |
| ZIMD v1 | Vial 7 | Moderna | 1 | 12 |
| ZIMD v1 | Vial 8 | Moderna | 2 | 12 |
| ZIMD v1 | Vial 9 | Moderna | 1 | 12 |
| ZIMD v1 | Vial 10 | Moderna | 2 | 11 |
| ZIMD v1 | Vial 11 | Moderna | 1 | 12 |
| ZIMD v1 | Vial 12 | Moderna | 1 | 12 |
| ZIMD v1 | Vial 13 | Moderna | 2 | 11 |
| ZIMD v1 | Vial 14 | Moderna | 1 | 12 |
| ZIMD v1 | Vial 15 | Moderna | 2 | 12 |
| ZIMD v1 | Vial 16 | Moderna | 2 | 12 |
| ZIMD v1 | Vial 17 | Moderna | 1 | 11 |
| ZIMD v1 | Vial 18 | Moderna | 1 | 12 |
| ZIMD v1 | Vial 19 | Moderna | 1 | 12 |
| ZIMD v1 | Vial 20 | Moderna | 2 | 12 |
| ZIMD v1 | Vial 21 | Moderna | 1 | 11 |
| ZIMD v1 | Vial 22 | Moderna | 2 | 12 |
| ZIMD v1 | Vial 23 | Moderna | 1 | 12 |
| ZIMD v1 | Vial 24 | Moderna | 2 | 11 |
| ZIMD v1 | Vial 25 | Moderna | 1 | 12 |
| ZIMD v1 | Vial 26 | Moderna | 2 | 12 |
| ZIMD v1 | Vial 27 | Moderna | 1 | 12 |
| ZIMD v1 | Vial 28 | Moderna | 2 | 12 |
| ZIMD v1 | Vial 29 | Moderna | 1 | 11 |
| ZIMD v1 | Vial 30 | Moderna | 2 | 12 |
| ZIMD v1 | Vial 31 | Moderna | 1 | 12 |
| ZIMD v1 | Vial 32 | Moderna | 2 | 11 |
| ZIMD v1 | Vial 33 | Moderna | 1 | 12 |
| ZIMD v1 | Vial 34 | Moderna | 2 | 12 |
| ZIMD v1 | Vial 35 | Moderna | 1 | 12 |
| ZIMD v1 | Vial 36 | Moderna | 2 | 12 |
| ZIMD v1 | Vial 37 | Moderna | 1 | 11 |
| ZIMD v1 | Vial 38 | Moderna | 2 | 12 |
| ZIMD v1 | Vial 39 | Moderna | 1 | 11 |
| ZIMD v1 | Vial 40 | Moderna | 2 | 12 |
| ZIMD v1 | Vial 41 | Moderna | 1 | 12 |
| ZIMD v1 | Vial 42 | Moderna | 2 | 11 |
| ZIMD v1 | Vial 43 | Moderna | 1 | 12 |
| ZIMD v1 | Vial 44 | Moderna | 2 | 12 |
| ZIMD v1 | Vial 45 | Moderna | 1 | 11 |
| ZIMD v1 | Vial 46 | Moderna | 2 | 12 |
| ZIMD v1 | Vial 47 | Moderna | 1 | 12 |
| ZIMD v1 | Vial 48 | Moderna | 2 | 11 |
| ZIMD v1 | Vial 49 | Moderna | 1 | 12 |
| ZIMD v1 | Vial 50 | Moderna | 2 | 11 |
| ZIMD v1 | Vial 51 | Moderna | 1 | 12 |
| ZIMD v1 | Vial 52 | Moderna | 2 | 12 |
| ZIMD v1 | Vial 53 | Moderna | 1 | 11 |
| ZIMD v1 | Vial 54 | Moderna | 2 | 12 |
| ZIMD v1 | Vial 55 | Moderna | 1 | 12 |
| ZIMD v1 | Vial 56 | Moderna | 2 | 11 |
| ZIMD v1 | Vial 57 | Moderna | 2 | 12 |
| ZIMD v1 | Vial 58 | Moderna | 1 | 12 |
| ZIMD v1 | Vial 59 | Moderna | 2 | 11 |
| ZIMD v1 | Vial 60 | Moderna | 2 | 12 |
| ZIMD v1 | Vial 61 | Moderna | 1 | 12 |
| ZIMD v1 | Vial 62 | Moderna | 2 | 11 |
| ZIMD v1 | Vial 63 | Moderna | 1 | 12 |
| ZIMD v1 | Vial 64 | Moderna | 2 | 12 |
| ZIMD v1 | Vial 65 | Moderna | 2 | 12 |
| ZIMD v1 | Vial 66 | Moderna | 1 | 11 |
| ZIMD v1 | Vial 67 | Moderna | 1 | 12 |
| ZIMD v1 | Vial 68 | Moderna | 2 | 11 |
| ZIMD v1 | Vial 69 | Moderna | 1 | 12 |
| ZIMD v1 | Vial 70 | Moderna | 2 | 12 |
| ZIMD v1 | Vial 71 | Moderna | 1 | 11 |
| ZIMD v1 | Vial 72 | Moderna | 1 | 12 |
| ZIMD v1 | Vial 73 | Moderna | 2 | 12 |
| ZIMD v1 | Vial 74 | Moderna | 1 | 11 |
| ZIMD v1 | Vial 75 | Moderna | 2 | 11 |
| ZIMD v1 | Vial 76 | Moderna | 1 | 12 |
| ZIMD v1 | Vial 77 | Moderna | 2 | 12 |
| ZIMD v1 | Vial 78 | Moderna | 1 | 12 |
| ZIMD v1 | Vial 79 | Moderna | 2 | 12 |
| ZIMD v1 | Vial 80 | Moderna | 1 | 11 |
| ZIMD v1 | Vial 81 | Moderna | 2 | 12 |
| ZIMD v1 | Vial 82 | Moderna | 1 | 12 |
| ZIMD v1 | Vial 83 | Moderna | 2 | 11 |
| ZIMD v1 | Vial 84 | Moderna | 1 | 12 |
| ZIMD v1 | Vial 85 | Moderna | 1 | 11 |
| ZIMD v1 | Vial 86 | Moderna | 2 | 11 |
| ZIMD v1 | Vial 87 | Moderna | 1 | 12 |
| ZIMD v1 | Vial 88 | Moderna | 2 | 12 |
| ZIMD v1 | Vial 89 | Moderna | 1 | 11 |
| ZIMD v1 | Vial 90 | Moderna | 2 | 12 |
| ZIMD v1 | Vial 91 | Moderna | 1 | 11 |
| ZIMD v1 | Vial 92 | Moderna | 2 | 12 |
| ZIMD v1 | Vial 93 | Moderna | 1 | 11 |
| ZIMD v1 | Vial 94 | Moderna | 2 | 12 |
| ZIMD v1 | Vial 95 | Moderna | 1 | 11 |
| ZIMD v1 | Vial 96 | Moderna | 2 | 11 |
| ZIMD v1 | Vial 97 | Moderna | 1 | 12 |
| ZIMD v1 | Vial 98 | Moderna | 2 | 11 |
| ZIMD v1 | Vial 99 | Moderna | 2 | 12 |
| ZIMD v1 | Vial 100 | Moderna | 2 | 11 |
| HPMT | Vial 1 | Moderna | 1 | 12 |
| HPMT | Vial 2 | Moderna | 1 | 12 |
| HPMT | Vial 3 | Moderna | 1 | 12 |
| HPMT | Vial 4 | Moderna | 2 | 12 |
| HPMT | Vial 5 | Moderna | 2 | 12 |
| HPMT | Vial 6 | Moderna | 1 | 12 |
| HPMT | Vial 7 | Moderna | 2 | 12 |
| HPMT | Vial 8 | Moderna | 1 | 12 |
| HPMT | Vial 9 | Moderna | 2 | 12 |
| HPMT | Vial 10 | Moderna | 1 | 12 |
| HPMT | Vial 11 | Moderna | 2 | 12 |
| HPMT | Vial 12 | Moderna | 1 | 12 |
| HPMT | Vial 13 | Moderna | 2 | 12 |
| HPMT | Vial 14 | Moderna | 2 | 12 |
| HPMT | Vial 15 | Moderna | 1 | 12 |
| HPMT | Vial 16 | Moderna | 2 | 12 |
| HPMT | Vial 17 | Moderna | 1 | 12 |
| HPMT | Vial 18 | Moderna | 2 | 12 |
| HPMT | Vial 19 | Moderna | 1 | 12 |
| HPMT | Vial 20 | Moderna | 2 | 12 |
| HPMT | Vial 21 | Moderna | 1 | 12 |
| HPMT | Vial 22 | Moderna | 2 | 12 |
| HPMT | Vial 23 | Moderna | 1 | 12 |
| HPMT | Vial 24 | Moderna | 1 | 12 |
| HPMT | Vial 25 | Moderna | 2 | 12 |
| HPMT | Vial 26 | Moderna | 1 | 12 |
| HPMT | Vial 27 | Moderna | 2 | 12 |
| HPMT | Vial 28 | Moderna | 1 | 12 |
| HPMT | Vial 29 | Moderna | 2 | 12 |
| HPMT | Vial 30 | Moderna | 1 | 12 |
| HPMT | Vial 31 | Moderna | 2 | 12 |
| HPMT | Vial 32 | Moderna | 1 | 12 |
| HPMT | Vial 33 | Moderna | 2 | 12 |
| HPMT | Vial 34 | Moderna | 1 | 12 |
| HPMT | Vial 35 | Moderna | 2 | 12 |
| HPMT | Vial 36 | Moderna | 1 | 12 |
| HPMT | Vial 37 | Moderna | 2 | 12 |
| HPMT | Vial 38 | Moderna | 1 | 12 |
| HPMT | Vial 39 | Moderna | 2 | 12 |
| HPMT | Vial 40 | Moderna | 1 | 12 |
| HPMT | Vial 41 | Moderna | 2 | 12 |
| HPMT | Vial 42 | Moderna | 1 | 12 |
| HPMT | Vial 43 | Moderna | 2 | 12 |
| HPMT | Vial 44 | Moderna | 1 | 12 |
| HPMT | Vial 45 | Moderna | 2 | 12 |
| HPMT | Vial 46 | Moderna | 1 | 12 |
| HPMT | Vial 47 | Moderna | 2 | 12 |
| HPMT | Vial 48 | Moderna | 1 | 12 |
| HPMT | Vial 49 | Moderna | 2 | 12 |
| HPMT | Vial 50 | Moderna | 1 | 12 |
| HPMT | Vial 51 | Moderna | 2 | 12 |
| HPMT | Vial 52 | Moderna | 1 | 12 |
| HPMT | Vial 53 | Moderna | 2 | 12 |
| HPMT | Vial 54 | Moderna | 1 | 12 |
| HPMT | Vial 55 | Moderna | 2 | 12 |
| HPMT | Vial 56 | Moderna | 1 | 12 |
| HPMT | Vial 57 | Moderna | 2 | 12 |
| HPMT | Vial 58 | Moderna | 1 | 12 |
| HPMT | Vial 59 | Moderna | 2 | 12 |
| HPMT | Vial 60 | Moderna | 1 | 12 |
| HPMT | Vial 61 | Moderna | 2 | 12 |
| HPMT | Vial 62 | Moderna | 1 | 12 |
| HPMT | Vial 63 | Moderna | 2 | 12 |
| HPMT | Vial 64 | Moderna | 1 | 12 |
| HPMT | Vial 65 | Moderna | 2 | 12 |
| HPMT | Vial 66 | Moderna | 1 | 12 |
| HPMT | Vial 67 | Moderna | 2 | 12 |
| HPMT | Vial 68 | Moderna | 1 | 12 |
| HPMT | Vial 69 | Moderna | 2 | 12 |
| HPMT | Vial 70 | Moderna | 2 | 12 |
| HPMT | Vial 71 | Moderna | 2 | 12 |
| HPMT | Vial 72 | Moderna | 1 | 12 |
| HPMT | Vial 73 | Moderna | 2 | 12 |
| HPMT | Vial 74 | Moderna | 1 | 12 |
| HPMT | Vial 75 | Moderna | 1 | 12 |
| HPMT | Vial 76 | Moderna | 1 | 12 |
| HPMT | Vial 77 | Moderna | 2 | 12 |
| HPMT | Vial 78 | Moderna | 1 | 12 |
| HPMT | Vial 79 | Moderna | 2 | 12 |
| HPMT | Vial 80 | Moderna | 1 | 12 |
| HPMT | Vial 81 | Moderna | 2 | 12 |
| HPMT | Vial 82 | Moderna | 1 | 12 |
| HPMT | Vial 83 | Moderna | 1 | 12 |
| HPMT | Vial 84 | Moderna | 2 | 12 |
| HPMT | Vial 85 | Moderna | 1 | 12 |
| HPMT | Vial 86 | Moderna | 2 | 12 |
| HPMT | Vial 87 | Moderna | 1 | 12 |
| HPMT | Vial 88 | Moderna | 2 | 12 |
| HPMT | Vial 89 | Moderna | 1 | 12 |
| HPMT | Vial 90 | Moderna | 2 | 12 |
| HPMT | Vial 91 | Moderna | 1 | 12 |
| HPMT | Vial 92 | Moderna | 2 | 12 |
| HPMT | Vial 93 | Moderna | 1 | 12 |
| HPMT | Vial 94 | Moderna | 2 | 12 |
| HPMT | Vial 95 | Moderna | 1 | 12 |
| HPMT | Vial 96 | Moderna | 2 | 12 |
| HPMT | Vial 97 | Moderna | 1 | 12 |
| HPMT | Vial 98 | Moderna | 2 | 12 |
| HPMT | Vial 99 | Moderna | 1 | 12 |
| HPMT | Vial 100 | Moderna | 2 | 12 |

**Table S2**: Actual value of volumes drawn per syringe to deliver a dose of 0.5 mL (NaCl 0.9%) as a function of the type of syringe (HPMT, ZIMED v1 or ZIMED v2). N=80 per syringe type.

| Syringe number | Syringue type | Dead volume (µl) |
| --- | --- | --- |
| 1 | HPMT | 2.660649 |
| 2 | HPMT | 1.97085111 |
| 3 | HPMT | 2.16793622 |
| 4 | HPMT | 1.18251067 |
| 5 | HPMT | 1.97085111 |
| 6 | HPMT | 2.75919156 |
| 7 | HPMT | 2.660649 |
| 8 | HPMT | 2.46356389 |
| 9 | HPMT | 1.37959578 |
| 10 | HPMT | 1.67522345 |
| 11 | HPMT | 3.44898945 |
| 12 | HPMT | 2.46356389 |
| 13 | HPMT | 1.47813833 |
| 14 | HPMT | 2.660649 |
| 15 | HPMT | 3.35044689 |
| 16 | HPMT | 1.18251067 |
| 17 | HPMT | 2.660649 |
| 18 | HPMT | 2.85773411 |
| 19 | HPMT | 2.46356389 |
| 20 | HPMT | 1.37959578 |
| 21 | HPMT | 3.15336178 |
| 22 | HPMT | 1.773766 |
| 23 | HPMT | 2.06939367 |
| 24 | HPMT | 2.26647878 |
| 25 | HPMT | 2.85773411 |
| 26 | HPMT | 2.95627667 |
| 27 | HPMT | 3.44898945 |
| 28 | HPMT | 2.16793622 |
| 29 | HPMT | 1.18251067 |
| 30 | HPMT | 2.06939367 |
| 31 | HPMT | 2.36502133 |
| 32 | HPMT | 2.660649 |
| 33 | HPMT | 3.25190433 |
| 34 | HPMT | 3.05481922 |
| 35 | HPMT | 2.46356389 |
| 36 | HPMT | 2.85773411 |
| 37 | HPMT | 3.547532 |
| 38 | HPMT | 3.547532 |
| 39 | HPMT | 3.15336178 |
| 40 | HPMT | 2.36502133 |
| 41 | HPMT | 3.15336178 |
| 42 | HPMT | 1.67522345 |
| 43 | HPMT | 2.95627667 |
| 44 | HPMT | 3.44898945 |
| 45 | HPMT | 3.05481922 |
| 46 | HPMT | 3.15336178 |
| 47 | HPMT | 2.56210645 |
| 48 | HPMT | 2.06939367 |
| 49 | HPMT | 2.56210645 |
| 50 | HPMT | 1.67522345 |
| 51 | HPMT | 3.44898945 |
| 52 | HPMT | 1.97085111 |
| 53 | HPMT | 2.95627667 |
| 54 | HPMT | 2.36502133 |
| 55 | HPMT | 2.660649 |
| 56 | HPMT | 1.08396811 |
| 57 | HPMT | 1.87230856 |
| 58 | HPMT | 2.56210645 |
| 59 | HPMT | 2.56210645 |
| 60 | HPMT | 2.95627667 |
| 61 | HPMT | 2.75919156 |
| 62 | HPMT | 1.87230856 |
| 63 | HPMT | 2.26647878 |
| 64 | HPMT | 2.36502133 |
| 65 | HPMT | 2.75919156 |
| 66 | HPMT | 1.67522345 |
| 67 | HPMT | 2.46356389 |
| 68 | HPMT | 1.37959578 |
| 69 | HPMT | 2.26647878 |
| 70 | HPMT | 1.87230856 |
| 71 | HPMT | 2.26647878 |
| 72 | HPMT | 2.16793622 |
| 73 | HPMT | 3.64607456 |
| 74 | HPMT | 2.46356389 |
| 75 | HPMT | 3.05481922 |
| 76 | HPMT | 2.16793622 |
| 77 | HPMT | 2.75919156 |
| 78 | HPMT | 2.46356389 |
| 79 | HPMT | 1.87230856 |
| 80 | HPMT | 2.16793622 |
| 1 | ZIMD v1 | 1.18251067 |
| 2 | ZIMD v1 | 0.29562767 |
| 3 | ZIMD v1 | 2.36502133 |
| 4 | ZIMD v1 | 3.84315967 |
| 5 | ZIMD v1 | 1.67522345 |
| 6 | ZIMD v1 | 0.09854256 |
| 7 | ZIMD v1 | 6.10963845 |
| 8 | ZIMD v1 | 2.06939367 |
| 9 | ZIMD v1 | 1.67522345 |
| 10 | ZIMD v1 | 4.434415 |
| 11 | ZIMD v1 | 0.19708511 |
| 12 | ZIMD v1 | 0.59125533 |
| 13 | ZIMD v1 | 2.75919156 |
| 14 | ZIMD v1 | 1.37959578 |
| 15 | ZIMD v1 | 5.71546822 |
| 16 | ZIMD v1 | 0.886883 |
| 17 | ZIMD v1 | 3.25190433 |
| 18 | ZIMD v1 | 0.29562767 |
| 19 | ZIMD v1 | 2.56210645 |
| 20 | ZIMD v1 | 2.06939367 |
| 21 | ZIMD v1 | 3.35044689 |
| 22 | ZIMD v1 | 3.05481922 |
| 23 | ZIMD v1 | 2.36502133 |
| 24 | ZIMD v1 | 2.85773411 |
| 25 | ZIMD v1 | 3.547532 |
| 26 | ZIMD v1 | 2.75919156 |
| 27 | ZIMD v1 | 3.84315967 |
| 28 | ZIMD v1 | 2.16793622 |
| 29 | ZIMD v1 | 2.56210645 |
| 30 | ZIMD v1 | 3.15336178 |
| 31 | ZIMD v1 | 1.87230856 |
| 32 | ZIMD v1 | 2.06939367 |
| 33 | ZIMD v1 | 2.56210645 |
| 34 | ZIMD v1 | 3.15336178 |
| 35 | ZIMD v1 | 2.46356389 |
| 36 | ZIMD v1 | 4.13878734 |
| 37 | ZIMD v1 | 2.56210645 |
| 38 | ZIMD v1 | 3.15336178 |
| 39 | ZIMD v1 | 2.56210645 |
| 40 | ZIMD v1 | 2.26647878 |
| 41 | ZIMD v1 | 1.97085111 |
| 42 | ZIMD v1 | 2.26647878 |
| 43 | ZIMD v1 | 5.41984056 |
| 44 | ZIMD v1 | 5.51838311 |
| 45 | ZIMD v1 | 4.53295756 |
| 46 | ZIMD v1 | 1.67522345 |
| 47 | ZIMD v1 | 2.95627667 |
| 48 | ZIMD v1 | 5.51838311 |
| 49 | ZIMD v1 | 2.85773411 |
| 50 | ZIMD v1 | 2.36502133 |
| 51 | ZIMD v1 | 3.64607456 |
| 52 | ZIMD v1 | 3.84315967 |
| 53 | ZIMD v1 | 2.06939367 |
| 54 | ZIMD v1 | 2.75919156 |
| 55 | ZIMD v1 | 2.75919156 |
| 56 | ZIMD v1 | 2.85773411 |
| 57 | ZIMD v1 | 2.26647878 |
| 58 | ZIMD v1 | 2.660649 |
| 59 | ZIMD v1 | 2.36502133 |
| 60 | ZIMD v1 | 1.773766 |
| 61 | ZIMD v1 | 2.660649 |
| 62 | ZIMD v1 | 1.87230856 |
| 63 | ZIMD v1 | 3.25190433 |
| 64 | ZIMD v1 | 2.16793622 |
| 65 | ZIMD v1 | 4.33587245 |
| 66 | ZIMD v1 | 3.84315967 |
| 67 | ZIMD v1 | 3.547532 |
| 68 | ZIMD v1 | 3.44898945 |
| 69 | ZIMD v1 | 5.12421289 |
| 70 | ZIMD v1 | 4.33587245 |
| 71 | ZIMD v1 | 5.02567034 |
| 72 | ZIMD v1 | 5.91255334 |
| 73 | ZIMD v1 | 3.25190433 |
| 74 | ZIMD v1 | 3.15336178 |
| 75 | ZIMD v1 | 2.26647878 |
| 76 | ZIMD v1 | 2.36502133 |
| 77 | ZIMD v1 | 6.70089378 |
| 78 | ZIMD v1 | 3.44898945 |
| 79 | ZIMD v1 | 0.886883 |
| 80 | ZIMD v1 | 6.89797889 |
| 1 | ZIMD v2 | 6.30672356 |
| 2 | ZIMD v2 | 3.74461711 |
| 3 | ZIMD v2 | 4.73004267 |
| 4 | ZIMD v2 | 5.41984056 |
| 5 | ZIMD v2 | 3.94170222 |
| 6 | ZIMD v2 | 4.82858522 |
| 7 | ZIMD v2 | 6.40526611 |
| 8 | ZIMD v2 | 4.82858522 |
| 9 | ZIMD v2 | 4.53295756 |
| 10 | ZIMD v2 | 4.92712778 |
| 11 | ZIMD v2 | 4.63150011 |
| 12 | ZIMD v2 | 5.41984056 |
| 13 | ZIMD v2 | 6.50380867 |
| 14 | ZIMD v2 | 4.04024478 |
| 15 | ZIMD v2 | 5.61692567 |
| 16 | ZIMD v2 | 2.75919156 |
| 17 | ZIMD v2 | 5.61692567 |
| 18 | ZIMD v2 | 4.04024478 |
| 19 | ZIMD v2 | 4.13878734 |
| 20 | ZIMD v2 | 3.547532 |
| 21 | ZIMD v2 | 2.56210645 |
| 22 | ZIMD v2 | 4.33587245 |
| 23 | ZIMD v2 | 5.12421289 |
| 24 | ZIMD v2 | 2.46356389 |
| 25 | ZIMD v2 | 7.981947 |
| 26 | ZIMD v2 | 4.434415 |
| 27 | ZIMD v2 | 4.33587245 |
| 28 | ZIMD v2 | 3.44898945 |
| 29 | ZIMD v2 | 5.22275545 |
| 30 | ZIMD v2 | 7.78486189 |
| 31 | ZIMD v2 | 4.92712778 |
| 32 | ZIMD v2 | 2.16793622 |
| 33 | ZIMD v2 | 2.26647878 |
| 34 | ZIMD v2 | 3.05481922 |
| 35 | ZIMD v2 | 5.61692567 |
| 36 | ZIMD v2 | 4.63150011 |
| 37 | ZIMD v2 | 5.41984056 |
| 38 | ZIMD v2 | 4.23732989 |
| 39 | ZIMD v2 | 7.48923423 |
| 40 | ZIMD v2 | 3.25190433 |
| 41 | ZIMD v2 | 2.16793622 |
| 42 | ZIMD v2 | 6.10963845 |
| 43 | ZIMD v2 | 2.85773411 |
| 44 | ZIMD v2 | 3.74461711 |
| 45 | ZIMD v2 | 7.39069167 |
| 46 | ZIMD v2 | 3.74461711 |
| 47 | ZIMD v2 | 8.57320234 |
| 48 | ZIMD v2 | 6.01109589 |
| 49 | ZIMD v2 | 8.67174489 |
| 50 | ZIMD v2 | 4.434415 |
| 51 | ZIMD v2 | 5.91255334 |
| 52 | ZIMD v2 | 7.29214911 |
| 53 | ZIMD v2 | 6.89797889 |
| 54 | ZIMD v2 | 2.56210645 |
| 55 | ZIMD v2 | 5.12421289 |
| 56 | ZIMD v2 | 6.40526611 |
| 57 | ZIMD v2 | 4.92712778 |
| 58 | ZIMD v2 | 4.53295756 |
| 59 | ZIMD v2 | 4.434415 |
| 60 | ZIMD v2 | 6.40526611 |
| 61 | ZIMD v2 | 3.94170222 |
| 62 | ZIMD v2 | 4.23732989 |
| 63 | ZIMD v2 | 6.99652145 |
| 64 | ZIMD v2 | 5.41984056 |
| 65 | ZIMD v2 | 4.13878734 |
| 66 | ZIMD v2 | 3.94170222 |
| 67 | ZIMD v2 | 4.92712778 |
| 68 | ZIMD v2 | 4.73004267 |
| 69 | ZIMD v2 | 5.61692567 |
| 70 | ZIMD v2 | 2.16793622 |
| 71 | ZIMD v2 | 6.79943634 |
| 72 | ZIMD v2 | 5.41984056 |
| 73 | ZIMD v2 | 6.99652145 |
| 74 | ZIMD v2 | 9.55862789 |
| 75 | ZIMD v2 | 4.33587245 |
| 76 | ZIMD v2 | 3.64607456 |
| 77 | ZIMD v2 | 4.04024478 |
| 78 | ZIMD v2 | 4.434415 |
| 79 | ZIMD v2 | 5.61692567 |
| 80 | ZIMD v2 | 8.37611723 |
